# Dental Caries and Reflex Paralysis

**Published:** 1888-10

**Authors:** 


### Dental Caries and Reflex Paralysis.

The following cases came under treatment last July, 1887, and may be of interest to some members of the profession. Two women, ages respectively 28 and 27. The exciting cause in both were carious molar teeth in inferior maxillary, producing sudden weakness in the right arm, the weakness increasing gradually to a maximum, so that the arm could only by a great effort be adducted or raised, and disappearing abruptly on the removal of the teeth. In the second of the two cases the weakness had existed for three months, and disappeared in a few hours after extraction of the teeth. The theories as to the pathology of these cases I give as follows: Dr. Brown-Sequard's, that peripheral irritation of a sensory nerve produces anæmia of the cord and impairment of nutrition and function. Sir William Gull refutes this opinion in the Guy's Hospital Report 61. II "Lewisson's" inhibitory theory demonstrated by his experiments on rabbits; squeezing the kidney, uterus or intestines of a rabbit producing paraplegia, the paralysis disappearing at once when the pressure was removed. Prof. Charcot classifies "urinary paraplegias" in three classes: (1.) Paraplegia in which the spinal cord is the seat of an inflammation. (2.) Paraplegia invasion rapid, cessation sudden, spinal cord unaffected. (3.) Paraplegia depending upon direct propagation of morbid process from the bladder, along the nerves to the cord.

The second class Prof. Charcot calls "reflex paraplegia" and it is under this head I propose to include the two cases given above.

Sodium Fluosilicate as an Antiseptic.—Dr. Behrens, of Philadelphia, reports a series of experiments to test the comprative efficiency of corrosive sublimate, carbolic acid, and sodium fluosilicate (silico-fluoride.) At the end of two weeks it was found that infusions of beef, hay, etc., containing as much as 0.05 per cent. of corrosive sublimate contained bacteria, and later these developed in solutions containing 0.1 per cent. The solutions which contained less than two per cent. of carbolic acid, likewise developed bacteria within a month. The solutions to which sodium fluosicicate had been added in the proportion of 1:2000 remained, under similar conditions, perfectly free from micro-organisms. These results suggest a trial of the new antiseptic in bowel disorders dependent on fermentation. Its nonpoisonous character would give it a very great advantage over mercurial salts.

Oil of Peppermint as an Antiseptic.—Dr. W. Leonard Braddon, in the *Lancet* (March 17, 24), speaks in the most enthusiastic terms of the practical value of the oil of peppermint as an antiseptic agent. Solutions of putrescible material containing one part of the oil in 3,000 remained in good condition three weeks. Meat enveloped in gauze impregnated with it kept perfectly seven months, while carbolic gauze failed in the third week, and sublimate gauze on the second. For internal use the doctor prefers the oil of peppermint to all other microbicides, on account of its harmless character, and he has employed it with the most satisfactory results as an antiseptic in surgery. In diphtheria the undiluted oil may be locally applied, and there is reason to expect from its results that we have not been able heretofore to obtain from any antiseptic without danger of poisoning the patient. [Query: To what extent can menthol replace the oil of peppermint in these uses? Its established usefulness in catarrhal troubles seems to be evidence that its action is similar.—Ed.]

To Ebonize Wood.—(Fisher in *Ph. Ztsch. f. Russ.* The wood, e. g. cherry, is finished with fine sand-paper, and then treated with a solution of alum 10 parts, water 180 parts, glycerine 4 parts. When dry a solution is applied which has been prepared in the following manner: One part of logwood chips and ten of bruised nut-galls are boiled one hour in 100 parts of water, the loss by evaporation being supplied from time to time. To the filtered solution, heated to boiling, is added a solution of one part of copperas and one of crystallized acetate of copper in two parts of water under constant stirring. The solution is again filtered and one part of a neutral solution of indigo is added. When the wood is dry, it is treated with a solution made by dissolving one part of iron filings in the cold in ten parts of vinegar. When completely dry, the surface is to be well rubbed with a mixture of linseed oil four parts, oil of turpentine, one part.

